# Incidence of multiple *Herpesvirus *infection in HIV seropositive patients, a big concern for Eastern Indian scenario

**DOI:** 10.1186/1743-422X-7-147

**Published:** 2010-07-06

**Authors:** Nilanjan Chakraborty, Sohinee Bhattacharyya, Chandrav De, Anirban Mukherjee, Dwipayan Bhattacharya, Shantanu Santra, Rathindra N Sarkar, Dipanjan Banerjee, Shubhasish K Guha, Utpal K Datta, Sekhar Chakrabarti

**Affiliations:** 1 Virology Department, ICMR Virus Unit, ID & BG Hospital, GB4, 57 Dr. SC Banerjee Road Beliaghata, Kolkata-700 010, India; 2 Microbiology Division, National Institute of Cholera and Enteric Diseases P33 CIT Scheme-XM, Kolkata-700 010, India; 3 Department of Medicine, Calcutta Medical College and Hospital, 88 College Street, Kolkata-700 073, India; 4 Department of Medicine, APEX Clinics, Calcutta Medical College and Hospital, 88 College Street, Kolkata 700 073, India; 5 Department of Tropical Medicine, School of Tropical Medicine, 108 C.R Avenue, Kolkata- 700 073, India

## Abstract

**Background:**

Human immunodeficiency virus (HIV) infection is associated with an increased risk for human *herpes viruses *(HHVs) and their related diseases and they frequently cause disease deterioration and therapeutic failures. Methods for limiting the transmission of HHVs require a better understanding of the incidence and infectivity of oral HHVs in HIV-infected patients. This study was designed to determine the seroprevalence of human herpes viruses (CMV, HSV 2, EBV-1, VZV) antibodies and to evaluate their association with age, sex as well as other demographic and behavioral factors.

**Results:**

A study of 200 HIV positive patients from Eastern India attending the Calcutta Medical College Hospital, Kolkata, West Bengal, Apex Clinic, Calcutta Medical College Hospital and ART Center, School of Tropical Medicine, Kolkata, West Bengal was done. Serum samples were screened for antibodies to the respective viruses using the indirect ELISA in triplicates.

*CytoMegalo virus *(CMV), *Herpes Simplex virus *type 2 (HSV-2), *Varicella Zoster virus *(VZV), and *Epstein Barr virus *(EBV-1) were detected in 49%, 47%, 32.5%, and 26% respectively.

**Conclusion:**

This study has contributed baseline data and provided insights in viral OI and HIV co-infection in Eastern India. This would undoubtedly serve as a basis for further studies on this topic.

## Background

The HIV/AIDS is a global epidemic and approximately 40 million people are living with HIV/AIDS worldwide [1]. About 95% of all HIV/AIDS infected people are living in developing countries. It is estimated that India is currently harboring about 5.134 million HIV infected cases and comprises of 65% cases of Southeast Asia. The HIV/AIDS pandemic in India has extended beyond the common classification of high-risk groups and now is common among the general population [2,3]. The nation is indeed at the threshold of an exponential growth of this epidemic. Opportunistic Infections (OIs) have been recognized as common complications of HIV infection due to immune deficiency. OI is the main reason behind hospitalization and substantial morbidity in HIV infected patients [4]. It necessitates toxic and expensive therapies and reduces the expected life span of such patients. Virtually all HIV-related mortality is preceded by opportunistic infection [5]. OIs encompass a wide variety of microorganisms that produce fulminant infections in immunocompromised HIV seropositive patients. Viral pathogens causing OI evoke a spectrum of illness ranging from asymptomatic to fulminant diseases in HIV-infected individuals. Since the onset of the acquired immunodeficiency syndrome (AIDS) epidemic in 1980; human herpes viruses have resulted in many of the secondary manifestations of human immunodeficiency virus (HIV) infection such as Painful rash due to Painful rash caused by herpes zoster [6,7]. Almost 45 million people worldwide have been infected with HIV, and prior to highly active antiretroviral therapy (HAART), more than 75% of all HIV-infected individuals developed HHV-related symptoms. The advent of HAART has decreased the incidence of opportunistic HHV diseases and improved the survival capacity of those who received the therapy. Whether HAART has altered the rate of HHV reemergence from latency or the ability of HHVs to produce clinical manifestations is not established [8]. Clearly, HHV-related malignancies remain a significant problem for the HIV infected patients [9,10]. *Cytomegalovirus *(CMV), *Herpes Simplex virus *1 & 2 (HSV-1 & 2), *Vericella Zoster virus *(VZV), *Epstein Barr virus *(EBV are the common *herpesviruses *in Indian subcontinent responsible for viral OIs in HIV positive populations [11]. These *herpes viruses *are usually acquired in childhood or young adulthood, establish a state of asymptomatic latency, and may eventually reactivate to give clinical disease later in life or following an HIV induced decline in cell-mediated immune control. *Herpes simplex virus *type 1 (HSV1) and type 2 (HSV2) cause primary and recurrent oral, genital and rectal ulceration and occasionally disseminated visceral and CNS disease [12]. In HIV infected individuals, re-activation of VZV causes prolonged and severe manifestation of herpes zoster [7]. Retinitis is most frequent clinical manifestation of CMV though other manifestations like gastrointestinal disease, encephalitis and pneumonia may occur.

In India, especially in the eastern part (including West Bengal), limited information is available on opportunistic infections among HIV seropositive individuals. The relative frequencies of specific opportunistic diseases may vary in different countries and even in different areas within the same country [[13-15], and [16]]. Early diagnosis of opportunistic infections and prompt treatment definitely contribute to increased life expectancy among infected patients delaying the progression to AIDS [17]. In India, quite often the diagnosis of OI is made only on clinical signs and symptoms or when illness is quite advanced, and by then it may be polymicrobial in nature [18,19]. Determining the spectrum of OIs and the changing pattern over the years, in a given region requires adequate surveillance and good diagnostic services that are not available in many parts of the country [20]. There is paucity of reports about nature of etiological agents causing various clinical manifestations in HIV disease in India [21,22]. Hence, integrated investigative procedures are vital, especially in early stages of HIV infection. The clinical manifestations of HIV infection in India (like other developing countries) are diverse. Spectrum of OIs with which most of the patients present in the clinics, reflects a wide variety of other endemic diseases prevalent within each region.

Thus, the importance of viruses in engendering many of the secondary opportunistic illnesses (and several of the tumors) of AIDS warrants a survey of their disease manifestations and clinical management. Our objective in this study therefore was to determine the prevalence of antibodies to opportunistic viral antigens in HIV infected Indian population. We focused primarily on the four most prevalent viruses of the *herpesviridae *family found in Eastern Indian population [22]. This study is aimed at providing baseline data on the prevalence of various viral OIs as part of the preliminary investigation on the dynamics of viral opportunistic infections in immunocompromised population of India. The purposes of this study were to determine the frequency of the major viral opportunistic infections in HIV seropositive patients/AIDS patients from Eastern India and to determine the risk factors associated with OI at the time of diagnosis among these patients in order to promote a greater awareness and management of this modern day "plague" and its complications.

## Materials and methods

### Study site and patient recruitment

In our study, we reviewed 200 HIV/AIDS patients, admitted between January 2006 to November 2008 at Calcutta Medical College Hospital, Kolkata, West Bengal, Apex Clinic, Calcutta Medical College Hospital- a referral center for patients of HIV infection or AIDS, and ART Center, School of Tropical Medicine, for the detection of viral opportunistic infections. Their HIV status was confirmed by three ERS(Enzyme Linked Immunosorbent Assay [ELISA], Rapid, Simple), an ELISA(HIV ELISA, Rapid test)and Western Blot as recommended by the National Aids Control Organization (NACO), Ministry of Health and Family Welfare, Government of India. The admitted patients were referred to us because they presented symptoms related to HIV infection or symptoms of unknown origin such as prolonged fever. The study group comprised of 140 (70%) males and 60 (30%) females, with mean ages of 36 ± 16 and 35 ± 12 years respectively. The patients included in the study were from different states of Eastern India. Our observations included only periods of hospitalization; we did not investigate patients' records after their discharge from the hospital. There were 26 males and 5 females in the patient group.

### Investigation of risk factors

Written consent was obtained from all the patients and their complete and relevant demographic information including age, sex, ethnicity, residential history, education status, sexual behavior, drug abuse, and other risk factors was recorded. A medical history was obtained for each patient, and all patients received a full clinical examination. The diagnosis of the viral opportunistic infections was based exclusively on well defined clinical symptoms and the determination of specific antibodies in serum by ELISA. Data representing the patient groups in various risk factors as described in Table 1.

**Table 1:** The HIV-seropositive patient groups in various risk factors

Risk Behavior	Male	Female
Blood transfusion	13	6

IVD abuse	25	3

Heterosexual	82	39

Homophiles	20	12

**Occupation**		

Unemployed	12	46

Government Service	13	-

Non Government Service	115	14

**Deaths**	3	2

This study has been approved by appropriate human subject's research review board, by the Institutional Ethical Committee of ICMR Virus Unit, Kolkata, India.

### Laboratory examination and diagnosis of viral OIs

The viral OIs were primarily diagnosed by the common clinical manifestations. For the diagnosis of CMV, the primary clinical symptoms were pneumonia, retinitis (an infection of the eyes), blindness and gastrointestinal disease The common clinical features of CMV disease is retinitis (which usually presents as painless, gradual loss of vision, floaters) followed by oesophagitis (presents as dysphagia - difficulty in swallowing or Odynophagia - painful swallowing), colitis (presents as pain abdomen, bloody diarrhea, fever), pneumonitis (cough, breathlessness), encephalitis (altered mental status, convulsion, headache), radiculoneuropathy (weakness/paralysis of lower limbs, pain lower back, urinary retention) etc. Primary symptoms of HSV included persistent vesicular and ulcerative lesions of the oral and anogenital areas, often with extensive or deep ulcerations and blisters on or around the genitals or rectum. The blisters left tender ulcers (sores) that took two to four weeks to heal the first time they occurred. Other symptoms included tender tonsils covered with a whitish substance that made swallowing difficult or blisters present in the mouth. Primary diagnosis of EBV was based on the clinical symptoms of fever, sore throat and swollen lymph glands. The commonest clinical presentation of EBV disease in HIV positives is Oral hairy Leukoplakia (OHL). VZV was primarily diagnosed based on the clinical manifestations of severe headaches, backache, general malaise and fever accompanied by the typical exanthem (rash) of chickenpox. Other symptoms of VZV included painful oral lesions, vesicular rash, facial numbness and loss of hearing/ear pain. All the clinical manifestations were confirmed by ELISA test done in triplicates. The following diagnostic test kits were used for the assay of the opportunistic viruses:

#### Anti- CMV

Serum anti-CMV was determined by a commercially available test kit, CMV IgM, IgG ELISA Test kit supplied by EQUIPAR was used to detect antibodies against the CMV-IE1 and CMV-pp65mII.

#### Anti - EBV 1

Serum anti-EBV was determined by a commercially available test kit, EBV IgG, IgM ELISA Test kit supplied by Virotech/Germany was used to detect antibodies against the affinity-purified gp125 + p18 Peptide.

#### Anti- HSV 2

Serum anti- HSVwas determined by a commercially available test kit, HSV type1 IgM ELISA Test kit supplied by EQUIPAR was used to detect antibodies against the affinity-chromatographically purified recombinant antigen HSV-2.

#### Anti - VZV

Serum anti-VZV was determined by a commercially available test kit, VZV IgM, IgG, IgA ELISA Test kit supplied by Virotech/Germany was used to detect antibodies against the Ellen Strain antigen (ATCC).

### Evaluation of whole blood CD4+ Lymphocyte count

The CD4+ count of the HIV seropositive patients (n = 200) was done at the discretion of the treating physicians. The CD4+ T cell percentages and the CD4+ counts were estimated by FACS Calibur flow cytometer (Becton Dickinson, San Jose, Calif., USA). Dual color immunophenotyping was performed using standard whole blood methodology.

### Statistical analysis

The data was analyzed by the use of MINITAB Statistical software version 13.1. A regression model was fitted by using the data to analyze the effect of co-variates (i.e. age, sex, mode of infection, CD4+) on different response variable (i.e. different opportunistic infection). The Chi Square test of independence was used for statistical analysis and the Null hypothesis (H_0 _hypothesis) has also been tested. A *P*- value of > 0.05 was regarded as statistically significant. The mean, median, mode and standard deviation has also been done by using the same software. 95% Class Intervals and Kramer's V value, a measure of the strength of association among the levels of the row and column variables were calculated according to conventional methods. The purpose of the regression model and Chi Square test is to test the effect of different covariates on different response variables. Using the P-value method, one can conclude whether a given covariate has an effect on the response variable. From our data analysis, it is clear that age, sex, mode of transmission and CD4+ count (co-variates) have effects on the OIs (response variables) of our study subjects.

## Results

Of the 200 HIV/AIDS patients studied, the samples positive for CMV, HSV, VZV, and EBV are 98 (49%), 94 (47%) 52 (26%), and 65 (32.5%) respectively. The incidence of CMV was higher among males than females 59/39. HSV was also found to be more predominant in the males 63/31. The incidence of VZV and EBV was also found to be dominant in the male population, the dominancy being 46/19 and 31/21 respectively.

**Figure 1:**
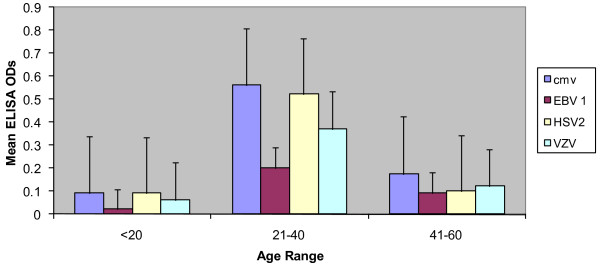
**Opportunistic viruses and CD4+ count in our patient cohorts**. The range of CD4+ counts/μL of blood are shown in index. The number of samples positive against the respective Opportunistic viruses are shown in bars corresponding to the CD4+ counts in the blood.

Age related prevalence of opportunistic viral antibodies in the serum of 200 HIV infected patient cohorts was assessed and results showed that individuals in the age group of 21- 40 years had the highest incidence of viral opportunistic infections as evident from Table. 2. The Figure 1 shows the respective opportunistic viral serum antibody incidence in the three age groups. The antibody OD with mean 0.275, CI (-0.34715, 0.897152); mean 0.237, CI (-0.373, 0.846336); mean 0.183, CI (-0.2251, 0.591771) mean 0.103, CI (-0.2192, 0.328658) for CMV, HSV, VZV and EBV respectively.

**Table 2:** The different opportunistic viruses which infects HIV/AIDS patients of different age groups

		Age Groups (in years)		
Opportunistic Viruses	Sex	≤ 20	21-40	41-60
CMV	Male	9	37	13
	
	Female	5	26	8

EBV	Male	-	22	9
	
	Female	2	15	4

HSV	Male	9	43	11
	
	Female	4	19	8

VZV	Male	9	24	13
	
	Female	2	10	7

Assessment of the risk factors associated with HIV transmission showed opportunistic viral incidence of HSV, CMV, VZV and EBV in Table. 3. HSV infection is found to be dominating in the heterosexual individuals among the study group. There are no significant variations among the homophiles, the drug users and the blood transfusion patients.

**Table 3:** Association of HIV transmission Risk factors with the various Opportunistic Viruses of the patients blood samples

	Risk Factors of HIV transmission							
	
	Heterosexual		Homophile		IDU		Blood transfusion	
	
Opportunistic Viruses	Male	Female	Male	Female	Male	Female	Male	Female
CMV	27	19	13	8	13	2	9	3

EBV	23	13	5	2	7	-	2	-

HSV	43	12	11	6	9	3	7	3

VZV	19	9	13	5	11	-	6	2

The incidence of the different viral OIs was also assessed with respect to the CD4+ cell count/μl of blood and patients with mean CD4+ cell count of 51-100 showed the highest prevalence of opportunistic viral antibodies. This was followed by the group with CD4+ count of 101-150, 51-100, 151-200 and >200 (data not shown).

## Discussion

Since the start of the epidemic, issues related to HIV/AIDS have had a high profile in industrialized countries. However, the burden of the disease continues to fall most heavily, and often less visibly in developing countries [23]. Viral opportunistic infections and HIV/AIDS having become so intertwined have constituted a major public health problem in the country. The opportunistic infections, therefore, play a major role in clinical presentations and remain one of the most frequent causes of death in these patients. However in spite of this, very little information on viral opportunistic infections and HIV co-infection in India is available. A few reports documented were only on HBV-HIV co-infection [24,25]. Viral opportunistic infections have not been given their desired attention in the Indian health care delivery system, largely due to the dearth of information on the co-infection of HIV positive population with viral OIs. Our study was therefore, designed to assess the incidence of HSV, EBV, CMV, and VZV infections among HIV patients in Eastern India so as to provide a baseline data on the dynamics of viral opportunistic infections in the immunocompromised population of Eastern India.

Infection by cytomegalovirus (CMV) is the major cause of morbidity and mortality in individuals with depressed cell mediated immunity of congenital origin, iatrogenic origin and that associated with acquired immunodeficiency syndrome (AIDS). The clinical diagnosis of AIDS with CMV infection can be difficult in the absence of CMV retinitis, polyradiculopathy and the classical CMV syndrome [26,27]. The diagnosis poses difficulties because a 2-3 week period is mandatory for virus isolation. While IgM antibodies as detected by ELISA correlate poorly with the clinical status of CMV infection and facilities for culture are usually not available in most centers [28]. There are a few reports available of CMV infection in Indian patients with HIV/AIDS, which are based primarily on clinical or autopsy evaluation [25,29,30]. We found CMV as the most incidental co infection in HIV/AIDS patient with the overall incidence of 49%.

Human immunodeficiency virus (HIV) infection is associated with an increased risk for human herpesviruses (HHVs) and their related diseases. The incidence of HSV in human immunodeficiency virus (HIV)-seropositive patients has not been focused, with reports generally focusing on individual infection [31]. In this report, the serum prevalence of HSV is found to be higher in HIV-seropositive patients; the overall incidence is around 47%.

VZV infection, the overall incidence being 32.5% is the third most incidental coinfection in HIV seropositive patients. VZV is one of the common aetiological agents of viral retinitis. Neurological complications of the reactivation of VZV occur most frequently in elderly persons and immunocompromised patients [32]. Gray *et al. *reported VZV infection of the CNS in more than 4% of patients with AIDS examined at autopsy. In AIDS patients, VZV tends to reactivate from multiple dorsal root ganglia levels, and the disease is often disseminated.

EBV is the least prevalent among the four HHVs studied in our work which is 26% of the total. EBV has been identified as a co-factor in the pathogenesis of a significant proportion of HIV related lymphoproliferative disorders and in oral hairy leukoplakia [33]. However, only limited information exists on the status of EBV in the course of HIV infection and the extent of its interaction with HIV. There is also growing interest in the biological properties and pathogenic potential of the different EBV subtypes, EBV-1 and EBV-2. Serological findings and studies in saliva and blood have indicated a high incidence of EBV-2 infection in the course of HIV disease, but there is limited information regarding its significance [34-37].

**Figure 2:**
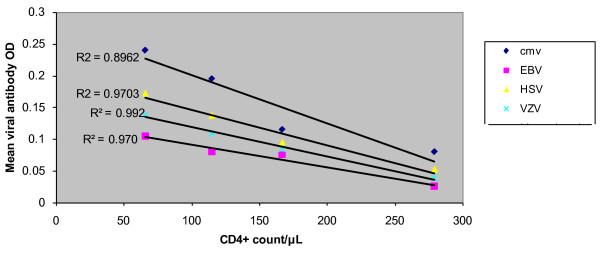
**CD4+ counts/μL of blood versus the antibody titres against the different opportunistic viruses in the patients**. The CD4+ count ranges between 50/μL to 300/μL and the antibody titres showing the mean OD of the triplicate ELISA reading.

**Figure 3:**
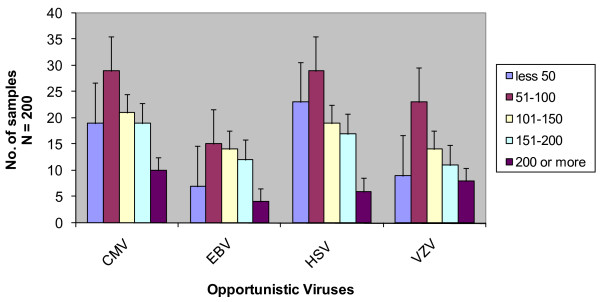
**Antibody prevalence of the corresponding viruses in different age groups of our patients samples**.

The analysis of the association of immunological status and the presence of viral OIs revealed that the CD4 count was significantly associated with the presence of viral OIs. An increasing CD4 count significantly protected patients from expressing HHVs in our patient cohorts as indicated by the correlation as in Figure 2. It has been detected clinically that more frequently virus infections are associated with the compromised immunity in HIV-infected patients [38-41]. We found that, HIV-infected patients with CD4+ cell counts of around 200 cells/mm3 are less likely to be infected with any virus examined here suggesting that a higherCD4+ cell count does play a role in immunity against virus infection. This clinical outcome is consistent with our finding of significantly lower CD4 cell count in HIV patients with viral OIs, indicating that the diagnosis of viral opportunistic infections can indeed be correlated with the clinical manifestation and thus is helpful in predicting disease progression. Figure 3 clearly shows that the patient group with CD4+ counts between 51 and 100 cells/μl is most susceptible to viral OI in HIV/AIDS patient. The groups with CD4+ count less than 50 cells/μl are showing least opportunistic viruses which could be due to the advanced HAART treatment.

This study is aimed at providing baseline data on viral opportunistic infections in HIV seropositive population as part of the preliminary investigation on the dynamics of viral opportunistic infections in immunocompromised population of India. One of the major problems is the lack of specific investigations that can provide rapid and reliable confirmation of a clinical diagnosis. A high level of alertness is needed at both clinical and laboratory level and routine surveillance studies need to be undertaken. Institutions in India and other developing countries need to be equipped to face the emerging challenge, in the form of updating the present knowledge, by way of education and training of the personnel, acquisition of skills of improved procedures, and their implementation in appropriate settings with adequate administrative support. Further investigations have to be undertaken with matched control samples as case control analysis with respect to all HHV infection in HIV seropositive individuals in Indian perspective.

## Competing interests

The authors declare that they have no competing interests.

## Authors' contributions

CD and SB have equal contribution to the work. NC conceived and designed the study. RNS, SS, DB, SKG and UD supplied the samples and did HIV testing. NC, CD, SB and AM carried out the laboratory investigations. CD, SB and NC analyzed and interpreted the data and drafted the manuscript. Dw Bh did the statistical analysis. SC monitored the total project. NC reviewed the manuscript critically for medical and intellectual content. All authors read and approved the final manuscript.
